# Food Swamps Predict Obesity Rates Better Than Food Deserts in the United States

**DOI:** 10.3390/ijerph14111366

**Published:** 2017-11-14

**Authors:** Kristen Cooksey-Stowers, Marlene B. Schwartz, Kelly D. Brownell

**Affiliations:** 1 Rudd Center for Food Policy and Obesity, University of Connecticut, Hartford, CT 06103, USA; marlene.schwartz@uconn.edu; 2 Sanford School of Public Policy, Duke University, Durham, NC 27708, USA; kelly.brownell@duke.edu

**Keywords:** food swamps, fast food retail, food deserts, food environments, obesity, instrumental variables, zoning

## Abstract

This paper investigates the effect of food environments, characterized as food swamps, on adult obesity rates. Food swamps have been described as areas with a high-density of establishments selling high-calorie fast food and junk food, relative to healthier food options. This study examines multiple ways of categorizing food environments as food swamps and food deserts, including alternate versions of the Retail Food Environment Index. We merged food outlet, sociodemographic and obesity data from the United States Department of Agriculture (USDA) Food Environment Atlas, the American Community Survey, and a commercial street reference dataset. We employed an instrumental variables (IV) strategy to correct for the endogeneity of food environments (i.e., that individuals self-select into neighborhoods and may consider food availability in their decision). Our results suggest that the presence of a food swamp is a stronger predictor of obesity rates than the absence of full-service grocery stores. We found, even after controlling for food desert effects, food swamps have a positive, statistically significant effect on adult obesity rates. All three food swamp measures indicated the same positive association, but reflected different magnitudes of the food swamp effect on rates of adult obesity (*p* values ranged from 0.00 to 0.16). Our adjustment for reverse causality, using an IV approach, revealed a stronger effect of food swamps than would have been obtained by naïve ordinary least squares (OLS) estimates. The food swamp effect was stronger in counties with greater income inequality (*p* < 0.05) and where residents are less mobile (*p* < 0.01). Based on these findings, local government policies such as zoning laws simultaneously restricting access to unhealthy food outlets and incentivizing healthy food retailers to locate in underserved neighborhoods warrant consideration as strategies to increase health equity.

## 1. Introduction

The nation is experiencing an obesity epidemic, with 35% of all adults classified as having obesity [1]. However, disparities exist and obesity rates are higher in low-income populations and racial and ethnic minority groups than in socially-advantaged populations [2]. There are also inequalities in obesity rates by geographic region, which has serious implications for exacerbating health disparities [3,4]. The relationship between where people live and their risk of obesity has led to research on the relationship between one’s food environment and health.

### 1.1. Food Deserts and Food Swamps

“Food deserts”, defined as residential areas with limited access to affordable and nutritious food, [5] have been posited as one driver of the obesity epidemic [6]. Living in a food desert has been linked to a poor diet [7] and greater risk of obesity [8,9]; while people who live near a grocery store are more likely to consume fruits and vegetables and less likely to be obese [6,10].

Food deserts are often assessed by measuring the distance between people’s homes and supermarkets [5,11], which has been found to vary significantly with a neighborhood’s racial/ethnic and socioeconomic composition [12,13,14]. To address the problem of food deserts, the Healthy Food Financing Initiative supports opening full-service grocery stores where none exist [15]. Surprisingly, quasi-experimental and longitudinal studies evaluating the impact of opening new grocery stores have shown that while perceived access to healthy food improves, diet quality and body mass index (BMI) do not [15,16,17,18]. These findings suggest that the influence of introducing healthier foods into a neighborhood may be tempered by the continued accessibility of unhealthy foods.

To capture the idea that both healthy and unhealthy food access is important, Rose and colleagues coined the term “food swamp” as a spatial metaphor to describe neighborhoods where fast food and junk food inundate healthy alternatives [19]. Low-income and racial-ethnic minorities are more likely than Whites to live near unhealthy food retailers, which has been associated with poor diet [20,21,22,23]. In a review of the research on fast food access, 10 out of 12 studies provided evidence that fast food restaurants are more likely to locate in areas where there are higher concentrations of ethnic minorities than Whites [24]. These associations raise questions about causality and suggest that the race and ethnicity of a community shapes the actions of the food industry and community design decision makers, which in turn, influence the food environment.

Alternatively, reverse causality may occur at the individual behavior level. Observational studies tend to assume that the food environment shapes individual health behaviors and health outcomes, but not vice versa. However, individuals may self-select into neighborhoods, and it is important that studies on neighborhood food environments and obesity account for this endogeneity problem [5,25,26,27,28,29,30,31,32,33]. Because it is not feasible to assign people to neighborhoods in an experimental design, statistical adjustments are necessary.

In summary, there is evidence that living in a food desert increases the risk of obesity. There is also emerging evidence that food swamps may better capture the characteristics of an obesogenic neighborhood food environment. The research to date on food swamps highlights two substantial gaps in knowledge: how to operationalize the food swamp concept for empirical analysis; and how to adjust for the possibility that obese adults choose to live in neighborhoods that are food swamps.

### 1.2. Study Objectives

To our knowledge, this is the first study to test relative measures of food swamps alongside food deserts as predictors of obesity rates across all United States (U.S.) counties, adjusting for reverse causality. We have three key objectives.

First, the present study makes a novel contribution by examining the countrywide existence of “food swamps” in the U.S. This builds upon previous work on food swamps abroad [33,34] and studies identifying food swamps in two major U.S. cities [21,35]. For instance, Hager and colleagues identified food swamps as areas in Baltimore City, with four or more convenience/corner stores within 0.25 miles of a study participant’s home.

Second, this study contributes to the literature on food swamps in the U.S. by considering the *relative balance* among multiple food outlets in the environment rather than absolute metrics of grocery store or restaurant access [36,37,38]. We examined multiple ways of categorizing food environments as food swamps, including alternate versions of the Retail Food Environment Index (RFEI) [23,34,39]. This builds upon previous work by Colón-Ramos and colleagues, which employed a relative food environment measure to identify a food swamp in the District of Columbia and predict food acquisition behaviors among recently migrated mothers from Central America [35]. The other studies that have used relative measures of food access were conducted outside the US, in Waterloo, Canada [33] and Porirua, New Zealand [34].

Third, this paper addresses the question of *reverse causality*, which is relevant because both individuals and the builders of food outlets choose to go into certain neighborhoods. Different statistical methods have been utilized to account for unobserved characteristics that may influence where individuals and food retailers choose to locate [5,26,27,28,29,30,31,32,33,40]. An increasingly common approach is to instrument for food store access with highways (counts within, or distance from, a predetermined geographic area), street connectivity or land zoned for commercial use [26,27,28,29].

To substantiate the use of highway exits as an instrument for restaurant access, previous papers have referenced the clustering of fast food retailers near highways, independent of demand or health outcomes [26,27,28,29,41]. With the exception of work by Dunn and colleagues using a nationally representative dataset, these studies have been limited to rural areas [26]. We expand upon this work by accounting for fast food retail options beyond the largest national brands and controlling for food deserts. We also include a measure of the conduciveness to physical activity to address the critical role of physical activity as a correlate of obesity [26].

Last, we offer an aggregated unit of analysis to facilitate policy discussions. We test the effect of food access on obesity rates by using a county-level unit of analysis [26]. This approach complements the current literature as it is more aggregated than the local community level [27,28,29,30,31,34,35,39], but less aggregated than the state level [32,42]. Previous work by Dunn [26] and Blanchard and Lyson [43] documents significant county-level variations in access to fast food restaurants or grocery stores. Land-use and zoning policies are usually implemented at the county and city-level; therefore, our county-level assessment of this topic can potentially inform an identification strategy for municipalities that would most benefit from revising zoning policies related to the local food environment [44].

The key hypotheses were that: (1) food swamps are a distinct and separate phenomenon to food deserts; and (2) food swamps have a stronger positive effect on obesity rates than food deserts, even after controlling for physical activity indicators and sociodemographic information.

## 2. Data and Empirical Strategy

We employed an instrumental variables (IV) strategy to correct for the endogeneity problems associated with food environments and utilized highway exits as a source of exogenous variation. Using cross-sectional, secondary food retail and obesity rates data from the USDA Food Environment Atlas, ordinary least squares (OLS) and two-stage least squares (2SLS) regression models were employed to analyze cross-sectional associations between local food environments categorized as food swamps and obesity rates.

### 2.1. Data

This paper used 2009 data from the United States Department of Agriculture (USDA) Food Environment Atlas [45]. This dataset includes 211 food environment indicators for all 3141 US counties. The Food Environment Atlas is a compilation of federal government sources and includes statistics on (1) food stores (total counts per county based on the North American Industry Classification System (NAICS); (2) health and well-being (% of adult county residents); and (3) community characteristics. Matching with county-level Federal Information Processing System (FIPS) codes, Food Environment Atlas data were combined with data from the ArcGIS ESRI StreetMap North America, on the number of highway exits per county as well as socio-demographic information from the American Community Survey.

### 2.2. Measures

#### 2.2.1. Dependent Variable: Adult Obesity Rates

The primary outcome measure was the rate of adult obesity at the county level, taken from the Food Environment Atlas, measured as the age-adjusted percentage of persons, aged 20 and older, who are obese. People are considered obese if they have a body mass index (BMI) greater than or equal to 30 kilograms per meter squared. BMI remains a respected screening tool for obesity, despite its limitations [46]. The original data source for this variable was the Centers for Disease Control and Prevention (CDC) Behavioral Risk Factor Surveillance Systems (BRFSS). CDC BRFSS calculates aggregate levels of BMI by asking more than 500,000 respondents nationwide the following questions: *How tall are you without shoes*? and *About how much do you weigh without shoes?*

#### 2.2.2. Independent Variable: Food Swamps

Table 1 presents the three continuous food swamp measures we used, which were based on previous studies utilizing relative food environment measures [23,30,33,34,39,47]. All county-level food store data was sourced from the Food Environment Atlas. We began with the traditional Retail Food Environment Index (RFEI), which is calculated as the ratio of fast food retailers and convenience stores to grocery stores and supermarkets [23,39]. The RFEI measure considers fast food restaurants, full-service restaurants, and convenience stores as unhealthy food outlets and supermarkets, grocery stores and farmers’ markets as healthy food outlets.

We also constructed two forms of “Expanded RFEI” to include additional food outlets. These additional outlets were categorized as healthy if they traditionally offered healthy foods, as defined in Table 2 [48]. Based on this definition, supermarkets, specialty stores (e.g., produce markets, delis), and permanent farmers markets were all classified as healthy outlets. Conversely, limited service establishments (which include take-out or self-carry to a table) and corner stores, and convenience stores were considered unhealthy outlets. Supercenters, such as Wal-Mart, were difficult to categorize because studies have found both positive and negative relationships between additional Wal-Mart locations and average BMI [49,50]. To address this empirically, we put superstores in the numerator (unhealthy) in RFEI #1 and the denominator (healthy) in RFEI #2.

### 2.3. Covariates

#### 2.3.1. Food Deserts

Food deserts were calculated as a continuous measure reflecting the proportion of each county’s total population identified as both low income and low access. Low income was defined as having a household income ≤ 200% of the federal poverty threshold and low access was defined as being more than 1 mile from a supermarket/grocery store in an urban area, or more than 10 miles in a rural area. The data for these calculations were obtained from the Food Environment Atlas.

#### 2.3.2. Recreation/Fitness Facilities & Natural Amenities

Ecological models of obesity illustrate energy balance as a function of factors shaping “energy in” (determined by the food environment), but also “energy out” (determined by the calories expended during physical activity). The Food Environment Atlas includes the number of recreation and fitness facilities that feature exercise, physical fitness and recreational sports unadjusted for county size (NAICS code: 713940). The original dataset for this variable was the U.S. Census Bureau County Business Patterns.

The Food Environment Atlas also provides a Natural Amenities Index for each county, originally from the Economic Research Service Natural Amenities Scale. The index is measured from 1 (low amenity) to 6 (high amenity) and is based on the premise that people are more inclined to be active where there are lakes, ponds or ocean fronts, warmer winters and summers with low-humidity.

#### 2.3.3. Socio-Demographic Characteristics

Matching on county-level FIPS codes, we merged data from the Food Environment Atlas (median household income, poverty rate, metropolitan vs. nonmetropolitan status and low-fat milk: soda price ratio) with data from the 2010 U.S. Census and the American Community Survey to obtain more information pertaining to neighborhoods characteristics. The low-fat milk price: soda price ratio variable was extracted from the Food Environment Atlas as a proxy for the relative affordability of healthier food products. More specifically, this measure is a ratio of the regional average price of 1% and nonfat milk to the regional average price of diet and caloric sweetened beverages. Variables taken from the Census included: population, number of households, race (% white, % Black and % Hispanic), gender (female), age groups (in four-year intervals) and size (in square miles).

Additional descriptive information about the counties, including educational attainment, means of travel to work (car, public transportation, etc.) and level of income equality (measured by the Gini Coefficient), was added from the American Community Survey. Previous evidence suggests that inequality is an important factor to consider in the context of obesogenic environments and health disparities [51]. The Gini Index reflects the distribution of income on a scale of 0 (perfect equality) to 1 (perfect inequality) [52]. Primary means of travel to work was included because transportation and vehicle ownership have been previously linked to food access [5]. All of these socio-demographic variables were measured at the county level, except for the low-fat milk: soda price ratio, which used regional prices.

### 2.4. Statistical Approach

Statistical analyses were executed using STATA/SE version 14 software (StataCorp, College Station, TX, USA). Initial descriptive statistics were calculated for the sample of 3141 US counties. Mean values were compared for the Food Store variables, the Food Environment Measures and key demographics, such as race, median household income, age, means of transport to work and Gini Index. The second phase of this study involved bivariate analyses. Pearson’s correlation coefficients were used to assess the relationships between obesity rates, highway exits, and food swamp measures. Correlational analyses were used to examine the association between the food swamp and food desert variables, and test the hypothesis that food swamps are operationally distinct from food deserts.

#### 2.4.1. OLS Regression Analysis: Testing Obesity as a Function of Food Swamps and Food Deserts

In phase three of the analyses, multivariate analyses were used to model county-level obesity rates as a function of the food swamp effect, the food desert effect, the number of recreation and physical fitness centers, and the Natural Amenities Index, while controlling for several neighborhood characteristics. Ordinary least squares (OLS) regression models were run to test the significance of these predictors. Each socio-demographic variable was added to the model only if its variance inflation factor (VIF) was about 2 or under. The model employed (Equation (1)) built upon one used by Chen and colleagues by incorporating food stores beyond fast food restaurants and grocery stores, and explicitly controlling for physical activity indicators in the aggregate (rather than at the individual) level [29].

County Obesity rate = *β*_0_ + *β*_1_ (*food swamp*) + *β*_2_ (*food desert*) + *β*_3_ (*# of recreation and physical fitness centers*) + *β*_4_ (*natural amenities*) + *γ* (*neighborhood characteristics*) + ϵ (1)

Each regression model was tested using three alternative food swamp measures. These were: (1) Traditional RFEI (2) Expanded RFEI with supercenters categorized as “healthy” and (3) Expanded RFEI 2 with supercenters categorized as “unhealthy”.

#### 2.4.2. Instrumental Variable Approach: Two-Staged Least Squared (2SLS) Regression Analyses

For the fourth phase of analysis, a quasi-experimental method was used to correct for reverse causality. One possibility is that unhealthy food outlets are built in neighborhoods where individuals already have expressed preferences for fast food and junk food, or are more likely to be obese. Therefore, to derive a consistent estimate of the effect of food swamps on obesity rates, we must be convinced that they are independent of residuals. To accomplish this, we used a two-stage least squares (2SLS) estimator for IV regression. The term “2SLS” stems from this estimator being calculated in two stages. The first stage regression is conducted using OLS and includes only exogenous variables on the right-hand side. The second stage involves utilizing OLS to estimate the structural regression, with endogenous variables replaced by predictors from the first stage. Rummo and colleagues employed comparable IV regression models to examine the relationship between food environments and body mass index [30].

Notably, the 2SLS IV approach requires that two conditions are met: (1) The chosen instrument is related to the potentially endogenous question predictor (X); and (2) There is no direct path from the instrument (Z) to the outcome (Y), except through predictor (X), known as the *exclusion restriction.*

The 2SLS IV approach employed in this study is illustrated by the results of two separate regressions (first and second stage). As shown in Equation (2), the first stage reflects Z (highway exits) as a valid predictor of food swamps (X). The second stage exhibits the predicted values of X (which were the output of the first stage) inserted into the model of county obesity rates (Y) (Equation (3)). We conducted a comparative analysis by running separate regression models, including only one of the three continuous food swamp measures. The “ivregress” command in Stata (version 15.0) was used for this phase of the analysis.

- First Stage Equation:
  Food swamps = *α*_0_ + *α*_1_ (*highway exit*) + *α*_2_ (*food desert*) + *α*_3_ (*recreation/fitnesscenters*) + *α*_4_ (*natural amenities*)+ *φ* (*neighborhood characteristics*) + *δ_i_* (2)
- Second Stage Equation:
  (3) $$\text{County Obesity rate}=\beta_{0}+\beta_{1}\left(\hat{food\;swamp}\right)+\beta_{2}\left(food\;desert\right)+\beta_{3}\left(recreation/fitnesscenters\right)\;+\beta_{4}\left(natural\;amenities\right)+\gamma \left(neighborhood\;characteristics\right)+\epsilon$$

#### 2.4.3. Stratification: Means of Travel to Work and Income Inequality

The final phase of the analysis involved stratification using the same models in Equations (1)–(3). As an attempt to isolate the food swamp effect on obesity rates from other county characteristics, counties included in the sampling frame were stratified by means of travel to work, and income equality. This permitted examination of the effect of food swamps on obesity rates, based on comparisons between more homogenous counties, while simultaneously addressing the potential threats to exclusion outlined above.

## 3. Results

### 3.1. Descriptive Statistics

Table 3 presents the descriptive statistics for the full sample of counties. Table 4 presents the number and percentage of counties considered food deserts or food swamps by each measure.

### 3.2. Bivariate Analysis

As Table 5 indicates, the relationship between food deserts and food swamps at the county level varied by the measure of food swamps used. Overall, this measure showed a statistically significant negative correlation between food deserts and food swamps. In addition, all of the coefficients reported in Table 5 were considered small, suggesting these two phenomena are distinctly different (see Appendix A, Table A2, for OLS estimates of food deserts on food swamps controlling for key socio-demographics). We found similar trends by testing the food desert measure (% low access and low income) as a predictor of all five food swamp variables, controlling for aggregate measures of educational attainment, race, ethnicity, poverty, and square miles

Results from the correlation analyses showed the food desert variable was not statistically significantly associated with obesity. In contrast, the food swamp measures were significantly positively correlated with the number of highway exits in a county. Surprisingly, the traditional RFEI was significantly negatively associated with the number of highway exits in a county. Finally, highway exits were significantly correlated with fitness centers. Physical activity indicators represent potential threats to the exclusion restriction, and thus were incorporated in the regression analyses (refer to Appendix A, Table A3, for correlations between obesity and highway exits and a range of food environment measures, physical activity indicators, and county demographics).

### 3.3. OLS Regression Results

All specifications included for the multivariate analysis used robust standard errors clustered by state (Table 6). The food swamp measures showed a statistically positive effect on obesity rates, controlling for food deserts, fitness/recreation centers, natural amenities, low-fat milk price: soda price ratio, county size in square miles and several sociodemographic indicators.

Across specifications (1–3) none of the continuous food desert measures remained statistically significant after controlling for food swamps. The magnitude of the food swamp effect on obesity varied across the three measures. However, the coefficients were small; the highest was the traditional RFEI variable, which indicated that a 1 percent increase in fast food restaurants increases obesity by approximately 0.125 percent. Here, the version of the expanded RFEI 1 with supercenters categorized as healthy had a larger magnitude than the expanded RFEI 2. However, neither had a stronger effect on obesity than the traditional RFEI. We also conducted the OLS regression analyses adding total food outlets as a covariate and there were no major changes in the findings (see Appendix A, Table A4, for the full table of regression results including total food stores as a neighborhood characteristic).

### 3.4. Instrument Variables (IV) Regression Results

In order to address the problem of food swamp endogeneity, we employed an IV strategy using the number of highway exits per county as a source of exogenous variation. The first and second stage results from the two-stage least squares (2SLS) regression analyses are shown in Table 7 and Table 8, respectively. The 2SLS models controlled for the same variables as the OLS regressions. Results from the first stage (Table 7) indicated that while highway exits were significantly associated with the food swamp measures, the size of the effect was small.

All food swamp measures indicated a positive effect on county-level obesity rates after controlling for food deserts, physical activity, and key demographics (Table 8). For instance, *β*_TraditionalRFEI_ was 2.604. Notably, using highway exits as an instrument, the food swamp effect on obesity was stronger compared to the OLS coefficients shown in Table 6. We also conducted these analyses controlling for total food outlets as a covariate in all models, and the food swamp measures still showed a positive relationship with obesity relevance. See the Appendix A Table A5 for the full table of IV results controlling for additional neighborhood characteristics and key demographics.

In the final analyses, the 2SLS model using the traditional RFEI was stratified by income inequality and means of transportation to work. As shown in Table 9, the food swamp effect remained significant among counties where there was less driving or reliance on public transportation for travelling to work (*β_RFEI_* = 2.452; *p* < 0.05). The first specification in Table 9 suggests food swamps are a stronger predictor of obesity in these geographic areas relative to the impact of food deserts (*β*_Lowincome&LowAccess_ = 0.09; *p* < 0.05). There were no statistically significant food swamp or food desert effects in counties with above average driving or use of public transportation. Further, the food swamp effect on obesity remained statistically significant in areas with lower income inequality (*p* < 0.05).

## 4. Discussion

This study documents that food swamps are a stronger predictor than food deserts on obesity rates among U.S. adults at the county level. Our results suggest that it is important to use relative food environment measures that incorporate both “healthy” and “unhealthy” retail sources of food. Our work expands the findings of a study conducted in California which showed a positive association between RFEI and obesity rates [23]. Our findings are also consistent with a previous longitudinal study of individuals’ accesses to grocery stores and fast food outlets which found evidence to support a 3 km zoning restriction on fast food establishments from low-income residents [40]. In that study, the authors concluded that increased access to grocery stores should be pursued in combination with other strategies, because improving access to grocery stores did not improve diet quality. The present study also demonstrates the value of addressing the potential problem of endogeneity when measuring the causal effect of food access on diet-related health outcomes. The current results demonstrate that typical OLS regression analyses would have underestimated both the food swamp and food desert effects on obesity rates.

### 4.1. Food Swamps vs. Food Deserts

The results of this study support the position that food swamps are a separate phenomenon from food deserts, and may play an even larger role than food deserts on county-level obesity rates. Results from OLS regressions showed that food desert measures became statistically insignificant after controlling for food swamps. In contrast, IV results indicated that food deserts have a significant positive effect on obesity. However, the food desert effect was much smaller than the food swamp effect on obesity after adjusting for sorting effects.

### 4.2. Food Swamp Measures

Overall, all the food swamp measures were significantly related to rates of adult obesity at the county level; however, the comparative analysis revealed variation in the magnitude of this effect across food swamp measures. We tested the traditional RFEI index and two expanded versions of the RFEI index and found that the traditional RFEI had the strongest relationship with rates of adult obesity out of the three measures, in both OLS and IV regression analyses. This suggests that the balance among fast food restaurants, convenience stores, and grocery stores is a more important determinant of aggregate obesity levels than other food outlets, including supercenters, farmers’ markets or specialized food stores.

### 4.3. OLS vs. IV

The present study also demonstrates the value of addressing the potential problem of endogeneity when measuring the causal effect of food access on diet-related health outcomes. Our results are consistent with previous studies showing that OLS regression models underestimate the effect of food environments on obesity rates [26,27,28,30,31,32]. Estimates from simple OLS regressions were downwardly biased and would have underestimated the food swamp effect without the highway exit instrument because they do not adjust for endogeneity. The naïve OLS estimate coefficients suggest that mitigating food swamp effects would only lower obesity rates by about 0.003 to 0.12 percent. In contrast, the IV coefficient estimates imply that policies, like zoning laws, could lower obesity rates by about three percent. These results are consistent with the notion that the built environment shapes health, even after controlling for selection and individuals’ preferences to live in certain neighborhoods [26,27,28,30,31,32,38,41,53,54].

### 4.4. Neighborhoods Characteristics

The positive food swamp effect on obesity rates was stronger in counties where people were less likely to drive or use public transportation to get to work. This finding suggests that individuals who have limited access to their own or public transportation may be more vulnerable to the negative impact of living in a food swamp. Zoning policies addressing food swamps might be prioritized in counties where people are restricted to walkable and bikeable distances when acquiring food (e.g., corner stores or fast-food restaurants near major roads).

In regard to the relative importance of transportation in food deserts, we found that in areas where the population was less mobile, living in a food swamp was more closely associated with rates of obesity. This result is consistent with the idea that grocery shopping decisions are less convenience driven than food purchasing at unhealthy food retailers. Thus, it is reasonable that food swamps are a stronger predictor in counties with comparable primary means of transportation, because people may consider proximity to fast food outlets and convenience stores to lower time costs associated with acquiring food. Notably, stratifying by income inequality did not diminish the food swamp effect, suggesting that this phenomenon is consistent in the built environment across socio-economically diverse counties.

### 4.5. Limitations

The present study has limitations. First, as noted by Dunn, highways are an imperfect instrument [26]. There were 1672 counties with no highway exits, so this IV approach did not provide information about counties where the food environment is not associated with the number of highway exits. Further, we might imagine several scenarios where the density of highway exits influences obesity rates through mechanisms other than “food swamp” environments. There are a few plausible threats to the exclusion restrictions necessary for implementing an IV approach. Gas stations selling snacks, quick meals and other high-calorie, convenience foods were not accounted for in the current study, but they also cluster near highways. Also, more highways may decrease the appeal of physical activity (i.e., walking or biking) due to more traffic congestion or pollution. We attempted to adjust for a potentially weak instrument [26,27,28] by controlling for physical activity indicators, median household income and race (threats 2 and 3). However, the physical activity indicators were measures of access and conduciveness to physical activity, not information about how frequently people exercise each week.

Second, due to data limitations, this study did not examine mechanisms linking food environments to obesity. It would be useful for future work on food swamps to consider diet quality, food away from home expenditures, and exposure to food marketing as potential pathways. Third, this study was limited to the county level data available, so it did not assess the impact of micro-level food environments by using census tract, neighborhood-level assessments, or individual-level BMI data. The cross-sectional nature of the dataset limited our ability to make causal statements, and the obesity rates were based on self-reported height and weight, rather than measured height and weight. Finally, there are validity issues with secondary data sources, including the Food Environment Atlas, which categorizes food stores based on NAICS codes [55].

## 5. Conclusions

### 5.1. Implications for Future Research on Zoning to Reduce Food Swamps

The findings from the current study have implications for zoning policies to reduce the harm associated with food swamps. Future research is needed to: (1) identify and refine the types of zoning policies recommended (e.g., restrictions on locations of fast food restaurants, closing times, distances from public places); (2) define terms such as “fast-food restaurant”, “formula restaurant”, and “carryout” to avoid enforcement challenges; (3) identify priority locations that meet the definition of food deserts or food swamps for zoning interventions; and (4) study how to mobilize community members and leaders.

Standardized measures of food swamps and fast food restaurants, in the context of health zoning policies, should be priority aims in future research on this topic. Fleischhacker and colleagues reported that twenty out of forty articles had their own way of defining a fast food retailer [55]. It is problematic that the Los Angeles City Council passed a moratorium on issuing licenses for the construction of new fast food restaurants without a sound definition of what constitutes a fast food restaurant [56].

### 5.2. Implications for Zoning as an Obesity Prevention Strategy

Scholars argue that policies limiting the availability or affordability of unhealthy foods may have more impact on obesity than those designed to promote access to healthy food options [14,57,58]. Given limited resources for policy interventions, it is important to identify which strategies are likely to have the greatest impact. However, the systems approach to obesity prevention has revealed that restricting unhealthy food items without introducing alternative healthy food stores potentially leads to higher rates of food insecurity in these communities [2].

The Institute of Medicine suggests that we identify ways to attract retailers offering healthier foods to underserved communities, while also limiting the concentration of unhealthy food venues. Incentives should be linked to public health goals in ways that give priority to stores that also commit to health-promoting retail strategies (e.g., through placement, promotion, and pricing) [2].

Results from the present study on food swamps can be used by key stakeholders, such as city planners and policymakers, to justify a two pronged approach to improving the food environment. Interventions like the Healthy Food Financing Initiative bring grocery stores to food deserts can be complemented with restrictions on fast-food restaurants and convenience stores. By simultaneously increasing availability of healthy food and decreasing availability of unhealthy food, policymakers can maximize the potential of the food environment to reduce obesity and promote health equity.

## Figures and Tables

**Table 1:** Food Swamp Measures.

Food Swamp Measure	Definition
Traditional Retail Food Environment Index (RFEI) [39]	$\frac{\mathrm{Fast}\;\mathrm{Food}/\mathrm{Limited}\;\mathrm{Service}\;\mathrm{Establishments}\;+\;\mathrm{Convenience}\;\mathrm{Stores}}{\mathrm{Grocery}\;\mathrm{Stores}/\mathrm{Supermarkets}}$
Expanded RFEI #1	$\frac{\mathrm{Fast}\;\mathrm{Food}/\mathrm{Limited}\;\mathrm{Service}\;\mathrm{Establishments}\;+\;\mathrm{Convenience}\;\mathrm{Stores}\;+\;\mathrm{Supercenters}}{\mathrm{Grocery}\;\mathrm{Stores}/\mathrm{Supermarkets}\;+\;\mathrm{Farme}r\prime \;s\;\mathrm{Markets}\;+\;\mathrm{Specialized}\;\mathrm{stores}}$
Expanded RFEI #2	$\frac{\mathrm{Fast}\;\mathrm{Food}/\mathrm{Limited}\;\mathrm{Service}\;\mathrm{Establishments}\;+\;\mathrm{Convenience}\;\mathrm{Stores}}{\mathrm{Grocery}\;\mathrm{Stores}/\mathrm{Supermarkets}\;+\;\mathrm{Farme}r\prime \;s\;\mathrm{Markets}\;+\;\mathrm{Specialized}\;\mathrm{stores}\;+\;\mathrm{Supercenters}}$

**Table 2:** Definitions of Healthy vs. Unhealthy Food Items [48].

Food Type	Definition
Healthy food	Foods that (a) are comprised of at least one of the major food groups (vegetables, fruits, grains, dairy, and protein foods) equal to at least half the portion size that the *Dietary Guidelines for Americans 2010* uses for measuring the nutrients in that food, and (b) contain only moderate amounts of saturated fats, added sugars, and sodium.
Less healthy/Unhealthy food	Foods that are high in saturated fat, added sugar, and/or sodium, or that contribute little to meeting dietary recommendations.

**Table 3:** Descriptive Statistics for all Counties in the United States (*N* = 3140).

Variable	Median (Standard Deviation)
Health	
Adult Obesity Rate (2009)	30.5 (4.16)
Physical Activity Measures	
Fitness Center ^†^	2.0 (30.31)
Natural Amenities Index (1 to 6) ^†^	3.0 (1.04)
Food Stores	
Fast Food Restaurants ^†^	15.0 (228.60)
Grocery Stores ^†^	6.0 (76.80)
Supercenters ^†^	0.0 (2.64)
Convenience Stores ^†^	16.0 (87.50)
Specialized Food Stores ^†^	1.0 (37.95)
Farmers Market ^†^	1.0 (4.00)
Food Environment Measures	
Food Desert (% Low income and Low Access to Grocery Store)	6.2 (8.37)
Food Swamp (Traditional RFEI)	3.5 (1.86)
Food Swamp (Expanded RFEI_1)	3.4 (2.14)
Food Swamp (Expanded RFEI_2)	3.6 (2.38)
Fast food retail per 10,000 ^†^	5.8 (2.99)
Number of fast food retailers ^†^	15.0 (228.58)
Demographics	
% Black	1.9 (14.42)
% Hispanic	3.3 (13.20)
Median Household Income ^†^	$41,255 (10,742)
% Female	50.4 (2.35)
Age over 65 ^†^	4000 (36,536.21)
Poverty Rate	15.9 (6.24)
Population ^†^	25,970 (313,819.30)
% Bachelor’s degree	12.2 (5.35)
% Drives to work	80.2 (7.79)
% Public transportation to work	0.4 (3.07)
% Walked to work	2.5 (3.75)
Gini Index ^†^	0.4 (0.04)
Other Key County Characteristics	
Highway exits ^†^	0.0 (33.51)
Low-Fat Milk: Soda Price Ratio ^†,^*	30.2 (62.10)
Square Miles ^†^	624.0 (1314.10)

Source: United States Department of Agriculture (USDA) Food Environment Atlas, U.S. Census 2010, American Community Survey. Notes: Expanded RFEI 1 and Expanded RFEI 2 include supercenters in the denominator and numerator, respectively. ^†^ Indicates variable is reported as the median. All other measures are presented as the median percentage. * Indicates variable is reported at the regional level.

**Table 4:** Food Environment Measures (*N* = 3108).

Food Environment Description	# of Counties
Expanded RFEI 1 (supercenters unhealthy) > 3.79	1470
Expanded RFEI 2 (supercenters healthy) > 4.02	1425
Traditional RFEI > 3.89	1419
Food Desert (% low income & low access) > 8.37	1193

Note: The cutoff values presented here are based on the mean value for each variable.

**Table 5:** Correlations between food swamp and food desert measures.

Food Swamp Measures	Food Desert Measures (% Low Access to Grocery Store and Low Income)
Expanded RFEI	−0.10 **
Expanded RFEI 2	−0.10 **
Traditional RFEI	−0.06 **

** Indicates *p* < 0.05.

**Table 6:** OLS estimates of food swamps on obesity, controlling for food desert effect for all U.S. counties (*N* = 3108) *.

Variables	1	2	3
Retail Food Environment Index (Modified version 1)	0.120 **		
	(0.0579)		
Retail Food Environment Index (Modified version 2)		0.115 **	
		(0.0499)	
Traditional RFEI			0.125 ***
			(0.0443)
% Low Income and Low Access	0.0149	0.0149	0.0138
	(0.0144)	(0.0144)	(0.0148)
Recreational Facilities	−0.00742	−0.00737	−0.00694
	(0.00454)	(0.00453)	(0.00453)
Natural Amenities	−1.049 ***	−1.049 ***	−1.039 ***
	(0.194)	(0.193)	(0.196)
Milk:Soda Price	1.484	1.480	1.501
	(1.658)	(1.656)	(1.670)
Constant	34.25 ***	34.26 ***	34.23 ***
	(1.568)	(1.563)	(1.573)
Observations	3069	3069	3055
R-squared	0.559	0.560	0.560

Robust standard errors in parentheses; *** *p* < 0.01, ** *p* < 0.05; All specifications control for number of fitness centers, natural amenities, % Black, % Hispanic, number of people over 65, poverty rate and county size (in square miles).

**Table 7:** Instrumental variables regression analysis, first-stage results (*N* = 3108).

Variables	RFEI Modified v1	RFEI Modified v2	Traditional RFEI
Highway exit coefficient	0.01	0.01	0.01
Highway exit *p* value	0.00	0.00	0.02
Instrument Test *F* test	7.87	7.44	15.66

Notes: In the first stage, the outcome was food swamps (a different measure of this phenomenon in each specification) and the number of highway exits in a county in the independent variable. All specifications control for % low income and low access (food desert measures), number of fitness centers, natural amenities, % Black, % Hispanic, number of people over 65, poverty rate and county size (in square miles).

**Table 8:** Coefficient estimates of food swamps on obesity rates using instrumental variable, *N* = 3108.

Variables	1	2	3
Retail Food Environment Index (Modified v1)	1.510 ***		
	(0.434)		
Retail Food Environment Index (Modified v2)		1.277 ***	
		(0.383)	
Traditional RFEI			2.604 **
			(1.076)
% Low Income and Low Access	0.0596 **	0.0540 **	0.0624 **
	−0.0235	−0.023	−0.0302
Recreational Facilities	0.000735	0.000188	0.0154
	−0.00438	−0.00438	−0.01
Natural Amenities	−1.177 ***	−1.165 ***	−1.212 ***
	−0.242	−0.231	−0.291
Low-Fat Milk:Soda Price	−1.202	−0.9	−2.797
	−1.779	−1.73	−2.237
Constant	32.31 ***	32.68 ***	28.57 ***
	−2.209	−2.09	−3.501
Observations	3069	3069	3055
Root Mean Square Error (RMSE)	3.95	3.81	5.21

Robust standard errors in parentheses; *** *p* < 0.01, ** *p* < 0.05. All specifications control for number of fitness centers, natural amenities, % Black, % Hispanic, number of people over 65, poverty rate and county size (in square miles).

**Table 9:** Instrument Variable (IV) coefficient estimates of food swamps on obesity rates (Models stratified by means of transportation to work & income inequality).

Variables	1	2	3	4
	Driving and Public Transportation Combined < 80%	Driving and Public Transportation Combined > 80%	Gini Coefficient < 0.41	Gini Coefficient > 0.41
Traditional RFEI	2.452 **	4.016	2.737 **	1.564 **
	(1.218)	(3.946)	(1.277)	(0.686)
Low Income and Low Access to Grocery Store	0.0932 **	−0.0794	0.0414	0.0690 *
	(0.0402)	(0.121)	(0.0279)	(0.0409)
Recreational Facilities	0.0131 *	0.0384	0.0178	0.0107
	(0.00773)	(0.0554)	(0.0112)	(0.0231)
Natural Amenities	−1.349 ***	−1.337 *	−1.235 ***	−1.100 ***
	(0.356)	(0.745)	(0.318)	(0.294)
Low-Fat Milk:Soda Price	−2.095	−4.081	−3.685	2.108
	(2.962)	(4.382)	(2.620)	(2.374)
Bachelor‘s Degree (%)	−0.266 ***	−0.288 ***	−0.269 ***	−0.253 ***
	(0.0426)	(0.0754)	(0.0456)	(0.0512)
Black (%)	0.0441 *	0.0391	0.0392	0.0204
	(0.0247)	(0.0515)	(0.0303)	(0.0357)
Hispanic (%)	−0.0649 ***	−0.0857 *	−0.0812 ***	−0.00185
	(0.0233)	(0.0440)	(0.0218)	(0.0185)
Poverty Rate (%)	0.0580	0.0891	0.0948 **	0.0685
	(0.0572)	(0.0691)	(0.0454)	(0.0447)
Square Miles	−9.09 × 10^5^	5.19 × 10^6^	−3.38 × 10^5^	−5.95 × 10^5^
	(0.000119)	(0.000433)	(0.000140)	(0.000123)
Constant	29.49 ***	24.97 **	29.00 ***	27.75 ***
	(3.395)	(10.25)	(3.461)	(3.706)
Observations	1230	1825	2442	613

Robust standard errors in parentheses; *** *p* < 0.01, ** *p* < 0.05, * *p* < 0.1.

## Appendix A

**Table A1:** Definitions for adult obesity rate & food environment variables.

Variable	Description	Data Source
Adult Obesity Rate	An estimate of the age-adjusted percentage of persons age 20 and older who are obese, where obesity is Body Mass Index (BMI) greater than or equal to 30 kilograms per meters squared.	Center for Disease Control
BUILT ENVIRONMENT		
Grocery Stores NAICS:445110	The number of supermarkets and grocery stores in the county. Grocery stores include establishments generally known as supermarkets and smaller grocery stores primarily engaged in retailing a general line of food, such as canned and frozen foods; fresh fruits and vegetables; and fresh and prepared meats, fish, and poultry.	U.S. Census Bureau, County Business Patterns
Specialized Food Stores NAICS 445200	The number of specialized food stores in the county. Specialized food stores include establishments primarily engaged in retailing specialized lines of food, such as retail bakeries, meat and seafood markets, dairy stores, and produce markets.	U.S. Census Bureau, County Business Patterns
Farmers Markets	The number of Farmers Markets in the county. A farmers’ market is a retail outlet in which two or more vendors sell agricultural products directly to customers through a common marketing channel. At least 51 percent of retail sales are direct to consumers.	USDA Agricultural Marketing Service
Supercenters NAICS:452910	The number of supercenters and warehouse club stores in the county. Warehouse clubs and supercenters are primarily engaged in retailing a general line of groceries in combination with general lines of new merchandise.	U.S. Census Bureau, County Business Patterns
Fast Food Restaurants NAICS:722211	The number of limited-service restaurants in the county. Limited-service restaurants include establishments primarily engaged in providing food services where patrons generally order or select items and pay before eating. Food and drink may be consumed on premises, taken out, or delivered to the customer’s location	U.S. Census Bureau, County Business Patterns
Convenience Stores/Food Marts NAICS:445120 and 447110	The number of convenience stores in the county. Establishments known as convenience stores or food marts are primarily engaged in retailing a limited line of goods that include soda, snack foods, etc.	U.S. Census Bureau, County Business Patterns

**Table A2:** OLS coefficient estimates of low income and low access (food desert measure) on three food swamp measures.

Variables	Modified RFEI (v1)	Modified RFEI (v2)	Traditional RFEI
% Low income and Low access	−0.0317 ***	−0.0330 ***	−0.0170 **
	(0.00924)	(0.0104)	(0.00831)
Bachelors degree (%)	0.000565	0.00515	−0.0375 ***
	(0.0111)	(0.0121)	(0.0108)
Black (%)	0.0283 ***	0.0295 ***	0.0143 ***
	(0.00646)	(0.00672)	(0.00437)
Hispanic (%)	0.0271 **	0.0293 **	0.0121
	(0.0115)	(0.0116)	(0.00942)
Poverty Rate	0.0139	0.0176	0.0113
	(0.0129)	(0.0148)	(0.0111)
Square Miles	−7.37 × 10^5^	−8.26 × 10^5^	−4.49 × 10^5^
	(6.53 × 10^5^)	(6.97 × 10^5^)	(4.66 × 10^5^)
Constant	3.427 ***	3.525 ***	4.167 ***
	(0.359)	(0.399)	(0.284)
Observations	3071	3071	3058
R-squared	0.078	0.072	0.047

Robust standard errors in parentheses; *** *p* < 0.01, ** *p* < 0.05.

**Table A3:** Correlations between obesity rates, highway exits, food environment measures, physical activity, and demographics.

Key Variables	Obesity	Highway Exits
Obesity		
Highway Exits	−0.20	
Physical Activity Measures		
Fitness Center	−0.29 *	0.84 *
Natural Amenities	−0.36 *	0.15 *
Food Stores		
Fast Food Restaurants	−0.22 *	0.86 *
Full Service Restaurants	−0.26 *	0.82 *
Grocery Stores	−0.20 *	0.74 *
Supercenters	−0.18 *	0.76 *
Convenience Stores	−0.18 *	0.86 *
Specialized Food Stores	−0.23 *	0.76 *
Farmers Market	−0.29 *	0.68 *
Food Environment Measures		
% Low Access to Grocery Store	0.024	−0.09 *
Traditional RFEI	0.16 *	−0.07 *
Expanded RFEI_1	0.11 *	0.04 *
Expanded RFEI_2	0.11 *	0.05 *
Demographics		
% Black	0.41 *	0.09 *
%Hispanic	−0.25 *	0.18 *
Median Household Income	−0.47 *	0.26 *
Poverty Rate	0.45 *	−0.08 *
Population	−0.21 *	0.86 *
% Bachelor’s degree	−0.57 *	0.32 *
% Drove to work	0.30 *	−0.02
%Public transportation to work	−0.24 *	0.30 *
% Walked to work	−0.18 *	−0.07 *
Gini Index	0.07 *	0.15 *
Other Key County Characteristics		
Gas stations with food	−0.16 *	0.85 *
Square Miles	−0.21 *	0.14 *
Area	−0.23 *	0.84 *
Metro	−0.14 *	0.33 *

* Indicates *p* < 0.05.

**Table A4:** OLS coefficient estimates of food swamps on obesity rates.

Variables	(1)	(2)	(3)	(4)	(5)
Food Swamp: Retail Food Environment Index (Modified v1)	0.118 **				
	(0.0582)				
Food Swamp: Retail Food Environment Index (Modified v2)		0.114 **			
		(0.0503)			
Food Swamp: Traditional RFEI			0.123 ***		
			(0.0450)		
Fast food retailers				0.00769 *	
				(0.00390)	
Fast food retailers/10,000 people					−0.0355 *
					(0.0177)
% Low income and Low Access	0.0151	0.0151	0.0140	0.00680	0.00775
	(0.0145)	(0.0145)	(0.0148)	(0.0131)	(0.0132)
Total Food Store	0.00148 **	0.00147 **	0.00149 **	0.00157 **	−0.00234
	(0.000569)	(0.000567)	(0.000569)	(0.000623)	(0.00216)
Recreational Facilities	−0.0273 ***	−0.0272 ***	−0.0269 ***	−0.0294 ***	−0.0331 ***
	(0.00796)	(0.00792)	(0.00795)	(0.00839)	(0.00797)
Natural Amenities	−1.043 ***	−1.043 ***	−1.033 ***	−1.038 ***	−1.049 ***
	(0.194)	(0.193)	(0.196)	(0.201)	(0.201)
Low-fat milk: Soda Price	1.653	1.648	1.670	1.936	2.250
	(1.670)	(1.668)	(1.683)	(1.724)	(1.758)
Bachelors degree (%)	−0.301 ***	−0.302 ***	−0.301 ***	−0.290 ***	−0.294 ***
	(0.0347)	(0.0348)	(0.0338)	(0.0357)	(0.0346)
Black (%)	0.0647 ***	0.0647 ***	0.0669 ***	0.0663 ***	0.0664 ***
	(0.0169)	(0.0170)	(0.0169)	(0.0169)	(0.0169)
Hispanic (%)	−0.0457 ***	−0.0458 ***	−0.0441 ***	−0.0422 ***	−0.0422 ***
	(0.0136)	(0.0137)	(0.0135)	(0.0135)	(0.0131)
Poverty Rate	0.101 ***	0.101 ***	0.0964 ***	0.105 ***	0.103 ***
	(0.0297)	(0.0298)	(0.0298)	(0.0310)	(0.0312)
Square Miles	−6.14 × 10^5^	−6.08 × 10^5^	−6.78 × 10^5^	−6.10 × 10^5^	−6.33 × 10^5^
	(8.40 × 10^5^)	(8.40 × 10^5^)	(8.41 × 10^5^)	(8.56 × 10^5^)	(8.65 × 10^5^)
Constant	34.06 ***	34.07 ***	34.03 ***	34.25 ***	33.94 ***
	(1.573)	(1.567)	(1.577)	(1.573)	(1.605)
Observations	3069	3069	3055	3108	3108
R-squared	0.561	0.562	0.562	0.559	0.561

Robust standard errors in parentheses; *** *p* < 0.01, ** *p* < 0.05, * *p* < 0.1.

**Table A5:** 2SLS (IV) coefficient estimates of food swamps on obesity rates.

Variables	(1)	(2)	(3)	(4)	(5)
Food Swamp: Retail Food Environment Index (Modified v1)	1.165 ***				
	(0.350)				
Food Swamp: Retail Food Environment Index (Modified v2)		0.993 ***			
		(0.317)			
Food Swamp: Traditional RFEI			2.074 **		
			(0.864)		
Fast food retailers				0.0952	
				(0.0812)	
Fast food retailers/10,000 people					2.973 *
					(1.595)
% Low income and Low Access	0.0487 **	0.0446 **	0.0522 **	−0.00416	0.176 *
	(0.0200)	(0.0200)	(0.0243)	(0.0194)	(0.0918)
Total Food Store	0.00117 ***	0.00114 ***	0.00105 *	−0.0465	−0.00107
	(0.000395)	(0.000394)	(0.000584)	(0.0432)	(0.00185)
Recreational Facilities	−0.0171 **	−0.0170**	−0.00340	−0.0803 **	0.00712
	(0.00695)	(0.00704)	(0.0119)	(0.0357)	(0.0300)
Natural Amenities	−1.140 ***	−1.132***	−1.171 ***	−1.104 ***	−1.566 ***
	(0.223)	(0.215)	(0.259)	(0.234)	(0.532)
Low-fat milk: Soda Price	−0.404	−0.191	−1.762	5.698 *	2.858
	(1.657)	(1.635)	(1.956)	(3.087)	(2.891)
Bachelors degree (%)	−0.315 ***	−0.319***	−0.268 ***	−0.262 ***	−0.865 **
	(0.0337)	(0.0338)	(0.0370)	(0.0513)	(0.352)
Black (%)	0.0397	0.0429	0.0444 *	0.0676 ***	0.0595 **
	(0.0279)	(0.0266)	(0.0264)	(0.0187)	(0.0282)
Hispanic (%)	−0.0711 ***	−0.0689***	−0.0663 ***	−0.0377 ***	−0.0696 ***
	(0.0151)	(0.0156)	(0.0179)	(0.0132)	(0.0202)
Poverty Rate	0.0936 ***	0.0917***	0.0854 **	0.0984 ***	−0.0472
	(0.0322)	(0.0330)	(0.0343)	(0.0337)	(0.122)
Square Miles	−1.82 × 10^5^	−1.81 × 10^5^	−5.35 × 10^5^	−9.71 × 10^5^	−5.04 × 10^5^
	(0.000107)	(0.000103)	(0.000109)	(9.50 × 10^5^)	(0.000247)
Constant	32.64 ***	32.92***	29.63 ***	31.47 ***	26.72 ***
	(1.972)	(1.891)	(3.008)	(2.263)	(4.658)
Observations	3069	3069	3055	3108	3108
R-squared	0.298	0.331		0.239	

Robust standard errors in parentheses; *** *p* < 0.01, ** *p* < 0.05, * *p* < 0.1.
